# The Slo3/Lrrc52 complex is sensitive to phosphoinositides

**DOI:** 10.1080/19336950.2020.1778393

**Published:** 2020-06-21

**Authors:** Takafumi Kawai, Yasushi Okamura

**Affiliations:** a Graduate School of Medicine, Osaka University, Suita, Japan; b Graduate School of Frontier Bioscience, Osaka University, Suita, Japan

**Keywords:** Slo3, Lrrc52, PIPs

## Abstract

The voltage-sensing phosphatase (VSP) is a unique protein that shows voltage-dependent phosphatase activity toward phosphoinositides. Recently, we reported that VSP is activated and generates polarized PtdIns(4,5)P_2_ distribution in sperm flagellum. Interestingly, such specialized PtdIns(4,5)P_2_ distribution appears to contribute to the activity of Slo3, a sperm-specific K^+^ channels. It has been already reported that Slo3 activity is upregulated by PtdIns(4,5)P_2_ using a heterologous expression system. However, PtdIns(4,5)P_2_-dependence of Slo3 activity has not been studied in heterologous expression system in the presence of auxiliary subunit of Slo3, Lrrc52, which drastically changes the electrophysiological property of Slo3. In the present study, we analyzed the regulation of Slo3 activity with Lrrc52 by VSP in *Xenopus* oocytes. Slo3 with Lrrc52 still exhibited similar sensitivity to VSP activity as Slo3 alone. This finding supports our previous report that VSP regulates Slo3 activity in native sperm flagellum.

## Main text

Phosphoinositides (PIPs) are important lipid molecules that are related to a variety of physiological processes [1]. Notably, many studies using heterologous expression system suggest that a substantial proportion of ion channels exhibit some sensitivity to PtdIns(4,5)P_2_ [2], suggesting that PtdIns(4,5)P_2_ may natively regulate the function of diverse ion channels. Recently, we discovered that a voltage-sensing phosphatase (VSP), which shows voltage-dependent phosphatase activity against PIPs [3], regulates the activity of PtdIns(4,5)P_2_-sensitive K^+^ channel, Slo3, by drastically reducing PtdIns(4,5)P_2_ level in sperm flagellum [4]. More interestingly, the extremely low level of PtdIns(4,5)P_2_ in sperm flagellum appears to be adjusted for the strong binding affinity of Slo3 to PtdIns(4,5)P_2_. The high PtdIns(4,5)P_2_-affinity of Slo3 was previously revealed by using water-soluble analog and scavenger of PtdIns(4,5)P_2_ [5]. In consistent with this report, using *Xenopus* oocyte expression system, we observed that Slo3 was sensitive but more tolerant to VSP activity than KCNQ2/3 which has low PtdIns(4,5)P_2_-affinity and is regulated by normal PtdIns(4,5)P_2_ level of plasma membrane [6].

**Figure 1.**
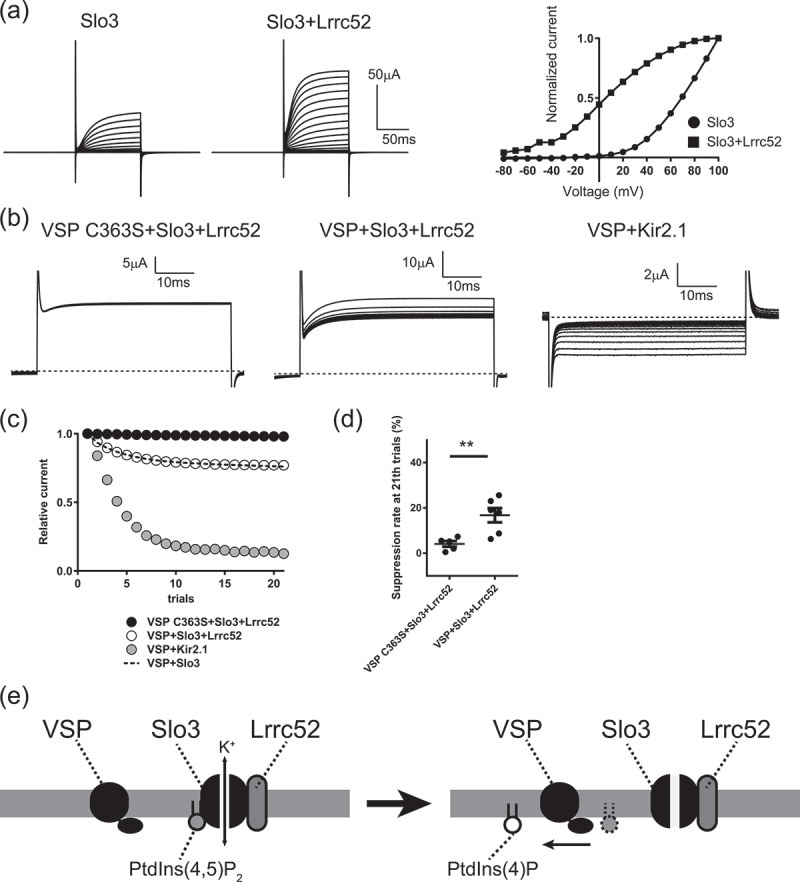
Functional coupling between Slo3/Lrrc52 complex with VSP reconstituted in *Xenopus* oocytes. (a) Lrrc52 drastically changes electrophysiological property of Slo3. Representative traces of Slo3 currents with or without Lrrc52. 100 ms voltage pulses in steps of 10 mV from −80 to +100 mV were applied. Holding potential was −60 mV. I–V relationships for Slo3 currents with (square) or without (circle) Lrrc52. (b) The representative traces of test pulses at +40 mV (Slo3) or −100 mV (Kir2.1) for 50 ms are shown. The test pulses were repeated for 21 times, and depolarization of +50 mV for 300 ms was applied in intervals between test pulses to activate Ci-VSP. The enzyme-defective mutant (Ci-VSP C363S) showed no change in current amplitude, indicating that the reduction of Slo3 or Kir2.1 current was due to enzyme activity of Ci-VSP. Dotted lines indicate the zero current level. (c) Representative data showing time course of relative current amplitude by repetitive VSP activation. The data for VSP+Slo3 was already reported in the previous paper and showed as broken line. (d) Statistical analysis showing inhibition percentage at 21th trials of test pulses. * indicates significant difference: p < 0.05, t-test. (e) The Slo3/Lrrc52 complex is sensitive to PIPs. The activity of Slo3/Lrrc52 complex is upregulated with strong binding of PtdIns(4,5)P_2_ (*Left*). After PtdIns(4,5)P_2_ is dephosphorylated by VSP in voltage-dependent manner, Slo3/Lrrc52 complex is less activated (*Right*).

However, the electrophysiological property of Slo3 expressed alone in heterologous expression system is known to be largely different from native Slo3 channel [7]. Lrrc52, which belongs to an extracellular leucine-rich-repeat-only (Elron) cluster, is one of the most important auxiliary subunits for Slo3 and drastically shifts the property of Slo3 to native function in sperm [7]. Therefore, it is important to address whether the strong binding affinity of Slo3 with PtdIns(4,5)P_2_ is maintained in the presence of Lrrc52. In the present study, we expressed Slo3 with Lrrc52 in *Xenopus* oocytes and examined the effect of *Ciona intestinalis* VSP (Ci-VSP) on the Slo3 channel activity. As previously reported [7], the co-expression of Lrrc52 with Slo3 largely shifted the I-V curve of Slo3 leftward (Figure 1(a)). Next, we tested the effect of activation of Ci-VSP on Slo3 (Figure 1(b)) [3]. The test pulses were repeated 21 times, and depolarization of +50 mV for 300 ms was applied in intervals between test pulses to activate Ci-VSP. When we expressed wild-type Ci-VSP with Slo3 and Lrrc52, the Slo3 current was gradually suppressed (Figure 1(b–d)). This reduction of Slo3 current was due to enzyme activity of Ci-VSP, because when we expressed Ci-VSP C363S, an enzyme-defective mutant [3], with Slo3 and Lrrc52, there was no remarkable change in current amplitude (Figure 1(b–d)). We also tested the effect of Ci-VSP activation on Kir2.1 that has higher affinity (Kd = 5 µM) to PtdIns(4,5)P_2_ than KCNQ2/3 (Kd = ~87 µM), but lower affinity than Slo3 (Kd = ~2.5 µM) as estimated by water-soluble analog of PtdIns(4,5)P_2_ [5,8]. At the 21st trial, Kir2.1 current was suppressed by 83.6 ± 3.0%, whereas Slo3 current with Lrrc52 was suppressed by 16.8 ± 3.1%. Thus, Slo3/Lrrc52 complex is more tolerant to VSP activity than Kir2.1, indicating that Slo3 still has a strong affinity to PtdIns(4,5)P_2_ in the presence of Lrrc52. Indeed, VSP-induced suppression of Slo3 current in the presence of Lrrc52 was as slow as that of Slo3 alone as already reported in our recent study [4] (Figure 1(c); The time constant was 3.905 ± 0.14, 3.5842 ± 0.0534, 3.384 ± 0.12 for VSP+Slo3, VSP+Slo3+Lrrc52, and VSP+Kir2.1, respectively, analyzed by single-exponential curve fitting). Furthermore, the percentage of inhibition in Slo3 current with Lrrc52 was comparable to that in Slo3 alone (15.1 ± 1.9%, Figure S10 in a previous paper [4]). Taken together with our recent findings, the activity of native Slo3 is regulated by extremely low concentration of PtdIns(4,5)P_2_ in sperm flagellum and affinity to PtdIns(4,5)P_2_ is adapted to subcellular distribution patterns of PtdIns(4,5)P_2_ [4].

## Materials and methods

### Two-electrode voltage clamp

The following cDNAs were used for cRNA synthesis for expression in *Xenopus* oocytes: *Ciona intestinalis*-VSP (Ci-VSP), Ci-VSP-C363S, mouse-Slo3 (mSlo3), mouse-Lrrc52 (mLRRC52), mouse-Kir2.1 in a custom modified version of the pcs2+; rat-Kv7.2 (KCNQ2) and rat-Kv7.3 (KCNQ3) in pGEM-HE were kind gifts from Drs. David McKinnon and Koichi Nakajo. For mSlo3 and mLRRC52, total RNA from the testis was prepared using TRIzol (Invitrogen, Waltham, MA, USA) and cDNA was generated using SuperScript® III (Invitrogen) according to the manufacturer’s protocol. Then, mSlo3 and mLRRC52 were cloned into pSD64TF. cRNA was synthesized using the mMESSAGE mMACHINE® Kit according to manufacturer’s protocol (Thermo Fisher Scientific). cRNA was injected into oocytes and the injected oocytes were incubated for 2–3 d at 18°C in ND96 solution containing (in mM): 5 HEPES, 96 NaCl, 2 KCl, 1.8 CaCl_2_, and 1 MgCl_2_ (pH 7.5), supplemented with gentamycin and pyruvate. Intracellular glass microelectrodes were filled with 3 M KCl. The microelectrode resistances ranged from 0.2 to 1.0 MΩ, and the ND96 solution was utilized as a bath solution. The macroscopic current was recorded under a two-electrode voltage clamp using an Oocyte Clamp amplifier (OC-725C: Warner Instruments, USA). Data acquisition was performed using Digidata1550A (Molecular Devices) and pCLAMP10.5 software (Molecular Devices).

### Data analysis

Data analysis was performed with Excel 2016 (Microsoft, USA), Clampfit 10.5 (Molecular Device, USA), and Igor Pro 6.37 (WaveMetrics, USA) software. Statistical analysis was performed with Prism 6 (GraphPad Software, San Diego, CA).
